# Pattern and Rate of Cognitive Decline in Cerebral Small Vessel Disease: A Prospective Study

**DOI:** 10.1371/journal.pone.0135523

**Published:** 2015-08-14

**Authors:** Andrew J. Lawrence, Rebecca L. Brookes, Eva A. Zeestraten, Thomas R. Barrick, Robin G. Morris, Hugh S. Markus

**Affiliations:** 1 Department of Clinical Neurosciences, University of Cambridge, Cambridge, United Kingdom; 2 Neurosciences Research Centre, Cardiovascular and Cell Sciences Research Institute, St George’s University of London, London, United Kingdom; 3 Department of Psychology, King's College Institute of Psychiatry, Psychology and Neurosciences, London, United Kingdom; INSERM U894, FRANCE

## Abstract

**Objectives:**

Cognitive impairment, predominantly affecting processing speed and executive function, is an important consequence of cerebral small vessel disease (SVD). To date, few longitudinal studies of cognition in SVD have been conducted. We determined the pattern and rate of cognitive decline in SVD and used the results to determine sample size calculations for clinical trials of interventions reducing cognitive decline.

**Methods:**

121 patients with MRI confirmed lacunar stroke and leukoaraiosis were enrolled into the prospective St George’s Cognition And Neuroimaging in Stroke (SCANS) study. Patients attended one baseline and three annual cognitive assessments providing 36 month follow-up data. Neuropsychological assessment comprised a battery of tests assessing working memory, long-term (episodic) memory, processing speed and executive function. We calculated annualized change in cognition for the 98 patients who completed at least two time-points.

**Results:**

Task performance was heterogeneous, but significant cognitive decline was found for the executive function index (p<0.007). Working memory and processing speed decreased numerically, but not significantly. The executive function composite score would require the smallest samples sizes for a treatment trial with an aim of halting decline, but this would still require over 2,000 patients per arm to detect a 30% difference with power of 0.8 over a three year follow-up.

**Conclusions:**

The pattern of cognitive decline seen in SVD over three years is consistent with the pattern of impairments at baseline. Rates of decline were slow and sample sizes would need to be large for clinical trials aimed at halting decline beyond initial diagnosis using cognitive scores as an outcome measure. This emphasizes the importance of more sensitive surrogate markers in this disease.

## Introduction

Cerebral small vessel disease (SVD) is the most common cause of vascular cognitive impairment and vascular dementia[1,2]. Cross sectional studies have shown that the cognitive profile in SVD is characterized by early impairments of processing speed and executive function with a relative sparing of episodic memory[3–5]. These neuropsychological deficits have a significant impact and are associated with poor functional outcome, such as a reduction in instrumental activities of daily living[6]. A number of cross-sectional studies[3–5] have investigated the pattern of cognitive impairment in SVD, but longitudinal reports of how cognitive function changes over time is rare. This information is important for providing prognosis to patients with SVD, monitoring the progress of disease, and is particularly vital for planning treatment trials. Important issues include understanding the rate of cognitive change and identifying tasks which provide the most sensitive and reliable measures of cognitive change in this population.

SVD describes a heterogeneous condition ranging from mild asymptomatic white matter hyperintensities (WMH)[7] seen in community populations, through patients with isolated lacunar stroke, to individuals with multiple lacunar infarcts and WMH who may suffer from vascular dementia[1]. A younger onset monogenic form of SVD, Cerebral Autosomal-Dominant Arteriopathy with Ischemic Leukoencephalopathy (CADASIL), also occurs[1]. The rate of cognitive decline varies between these phenotypes and therefore careful description of the patient group is important in any study of SVD. Furthermore, many patients defined by radiological SVD, particularly older patients who present to memory clinics, may also have coexistent Alzheimer’s pathology. To study a group with relatively pure vascular cognitive impairment, we recruited patients presenting to a stroke service who, regardless of cognitive complaints, had radiologically confirmed lacunar infarcts and leukoaraiosis. Previous studies have shown that the cross-sectional presentation of cognitive impairment in this group is similar to that seen in CADASIL patients[3] in whom cognitive impairment occurs at a younger age when co-existent Alzheimer’s disease pathology is not present.

We determined the rate and pattern of cognitive change over a 3 year follow-up period in a prospective cohort of patients with clinical lacunar stroke and leukoaraiosis. We used this information to perform power calculations for intervention trials and determined which cognitive domains would be most sensitive to change.

## Materials and Methods

### Ethics Statement

Study protocols were approved by a local research ethics committee (London—Wandsworth) and all patients provided prior written informed consent. The study is registered with the UK clinical research network (http://public.ukcrn.org.uk/, study ID: 4577).

### Participants

Participants were patients enrolled in the prospective St George's Cognition And Neuroimaging in Stroke (SCANS) study[5,8,9], a longitudinal investigation into the relationship between MRI markers and cognition in patients with symptomatic SVD. For this study we used cognitive data acquired at baseline and annually during the first three years of follow-up. Patients were recruited from inpatient and outpatient stroke services of three hospitals in South London, UK (St George’s, King’s College and St Thomas’ Hospitals) between 2007 and 2010 and followed up annually with cognitive assessment and MRI. SVD was defined as a clinical lacunar stroke syndrome[10] with an anatomically appropriate lacunar infarct on MRI, in addition to confluent leukoaraiosis (Fazekas grade ≥2)[11]. Exclusion criteria were: 1) any stroke mechanism other than SVD (extra or intracranial large artery stenosis >50%, cardioembolic source, non-lacunar subcortical infarcts >1.5cm in diameter as these are often caused by emboli, or cortical infarcts); 2) a history of major neurological or psychiatric disorders (with the exception of depression)[5]; 3) non-fluent in English, and; 4) unwilling or unable to undergo MRI. Patients who suffered a subsequent clinical stroke remained in the study provided the new stroke was lacunar and met the inclusion criteria as above. All patients were studied at least three months after their most recent stroke to reduce influences of acute ischemia on cognition.

### Neuropsychological Testing

Annually, patients underwent structured clinical examination, and completed a battery of widely used neuropsychological tasks chosen to characterize cognitive impairment in SVD[5,8]. Clinical assessment included the modified Rankin scale of disability and dependence following stroke[12]. Neuropsychological tasks are described in Table 1, and were grouped into four key cognitive domains. For each domain, task performance was evaluated on a common scale through the calculation of age-scale z-scores based on the best available published normative data[5,8] (see Table 1). Age scaling allows a meaningful average to be calculated within tasks related to a given domain. A Global Cognition measure of overall performance across all tasks was also produced by averaging all individual task scores. Parallel test forms were employed for two tests to reduce learning effects: the BMIPB Speed of Information Processing task (4 forms[13]) and single letter verbal fluency (annually alternating F-A-S and B-H-R). All other tasks were identical at each assessment. Premorbid IQ was measured using the National Adult Reading Test-Restandardized (NART-R)[14].

**Table 1:** Neuropsychological Test Battery.

**Cognitive Index**	**Task Name**	**Norms**	**Measure Description**	**Additional Details**
Working Memory	Digit Span[15]	[15]	Immediate recall of digit strings (forwards & backwards)	Total Score (sum of DS-F, DS-B)
Long Term Memory	Logical Memory[16]	[16]	Immediate and delayed recall of short stories	Total Score (sum of LM-I and LM-D)
	Visual Reproduction[16]	[16]	Immediate and delayed reproduction of line drawings	Total Score (sum of VR-I and VR-D)
Processing Speed	BMIPB SOIP[13]	[13]	Speeded cancellation of second highest of five two-digit numbers	Total Correct (adjusted for SOIP-M)
	Digit Symbol[15]	[15]	Speeded transcoding task	Total Correct
	Grooved Pegboard[17]	[18]	Pick-up, rotation and placement of small pegs.	Time Taken (best of L/R hand trials)
Executive Function	Trail Making Test[19]	[20]	Pen and paper sequencing task: alternating letters and numbers	Time to Complete Part B
	SL-Verbal Fluency[21]	[21]	Timed generation of words beginning with letter: FAS/BHR	Total Correct (all letters)
	mWCST[22]	[22]*	Card Sorting Test involving flexible shifting from learned dimensions.	Categories Completed & Perseverative Errors^+^
Global Cognition	All tasks listed above			

BMIPB—BIRT Memory and Information Processing Battery; DS-F—digit span (forwards); DS-B—digit span (backwards); LM-I—Logical Memory (Immediate); LM-D—Logical Memory (Delayed); VR-I—Visual Reproduction (Immediate); VR-D—Visual Reproduction (Delayed); SOIP-M—Speed of Information Processing Motor control task; mWCST—modified Wisconsin Card Sorting Test.

^*^The mWCST, selected because it is shorter yet retains the original task's lack of transition instructions, was scaled relative to a single published control group of comparable age and gender.

^+^The two task components were transformed to z-scores and a mean average composite mWCST score used. This composite mWCST measure is included in the global cognition index, not the separate sub-measures.

### Cognitive change

Annualized change scores were computed to allow the analysis of patients with partial data and to account for variability in the timing of assessments. For each subject, and each task measure, a linear regression was fitted to the data: *y* = *α* + *βx*, where *y* is the scaled cognitive score and *x* the time (in years) from baseline. The estimated parameters *α* and *β* therefore represent: the baseline score (*α*, the regression intercept) and the annualized change (*β*, the regression slope). The latter can be viewed as an extension of the commonly employed difference score method (final score − initial score). Mean-average annualized change scores were computed across tasks with non-missing data in each cognitive domain (WM, LTM, PS, and EF). Exploratory data analysis confirmed that trends over this timescale were well described by a linear fit (see S1 Table).

#### Poor performance in neuropsychological testing

For two tasks (trail making and grooved pegboard; Table 1) raw performance is measured as the time taken to complete the task. To reduce skew and the impact of extreme scores, performances where the age-scaled Z-score was less than −3.33 (corresponding to a scaled score <0) were recoded to this threshold value.

Floor effects, where a measure cannot discriminate performance below a certain point, are a particular concern for longitudinal studies of cognitive decline. When present, particularly at baseline, floor effects will reduce estimates of decline over time. To monitor this, we identified where subjects performed at the minimum possible level for each task, and by extension, cognitive indices where all the component tasks were performed at the minimum possible level.

### Sample size calculations

To assess implications for future research we produced 3-year difference scores (pro-rated from average annual change) and estimated the sample sizes required to detect treatment effects for both the cognitive indices and their constituent tasks. For example, if an average 3-year decline of 0.4 Z-units was observed on a measure, a treatment effect of 50% would be calculated based on the power to detect the difference between 0.4 in the control arm and 0.2 in the treatment arm given the observed standard deviation. We report sample size calculations based on 50, 40, 30 and 20 percent reductions in annual cognitive decline. Sample size calculations were based on a two-tailed, two-sample t-test with an alpha of 0.05 and a power of 0.80.

### Statistical Analysis

Analysis was carried out in R, version 3.02[23]. Analyses were corrected for multiple comparisons using the Holm-Bonferroni method. Annualized cognitive change was tested using one-sample, two-tailed t-tests (H_0:_ cognitive change = 0). Effects of cognitive index on cognitive change were further investigated using a repeated measures ANOVA predicting cognitive change from the within-patients factor: cognitive index (levels: WM, LTM, PS and EF), with and without demographic covariates (age at baseline, gender, and NART-IQ). To investigate the influence of baseline task performance on cognitive change, tests for correlation (Pearson’s r) were conducted.

#### Missing data

**Fig 1:**
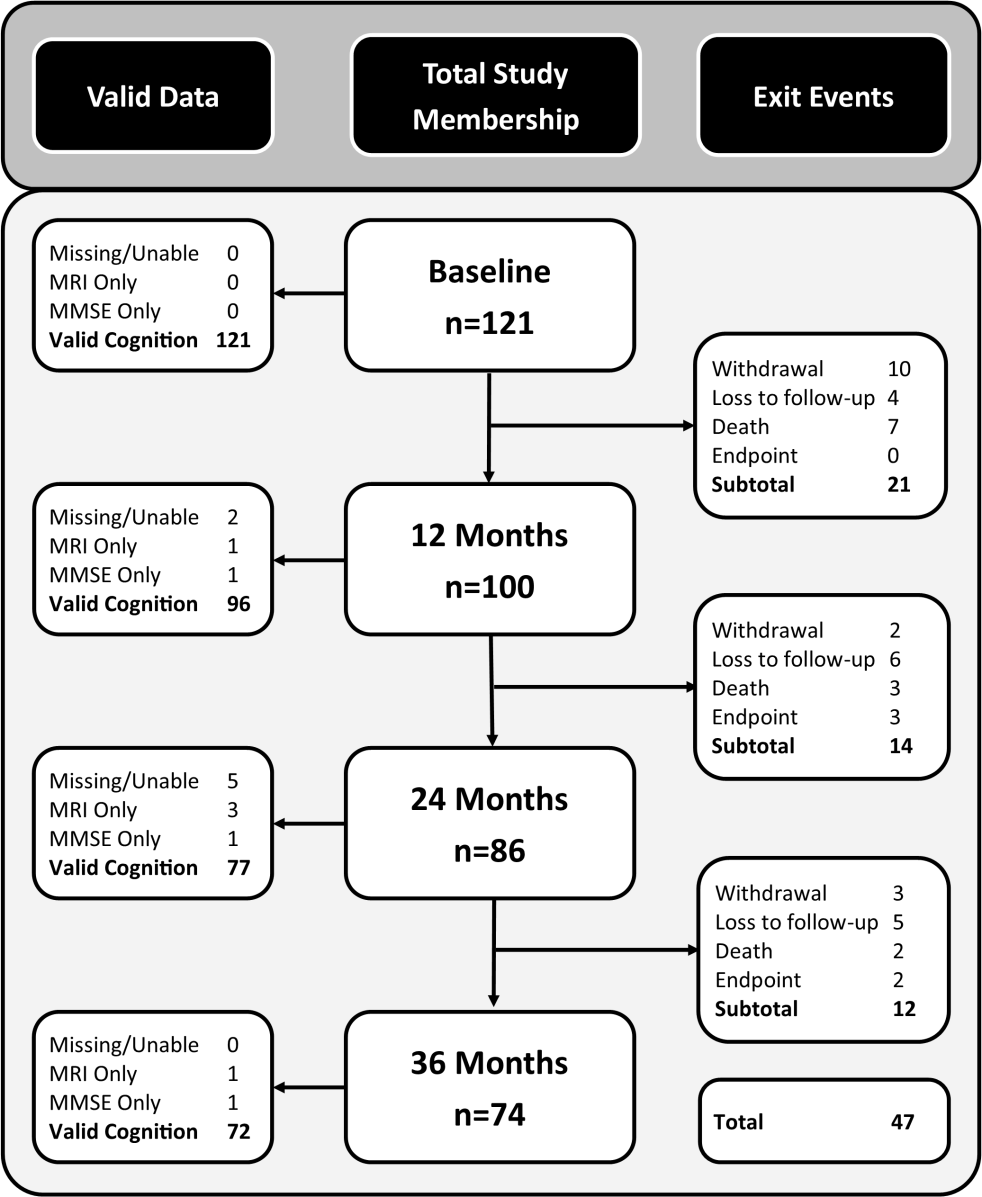
Flow diagram of study participant status. Middle column shows count data presented for total study membership; left shows valid neuropsychology assessment at each study time-point; and right are study exit events.

Missing data where a complete session was lost are described in Fig 1. There were also some sporadic missing data for individual tasks (3.04% across 9 tasks). Such missing data occurred when parts of the task battery were not completed on a given occasion. Reasons included: time constraints, patient motivation, experimenter error, and other task-specific factors which made data unsuitable. Such missing data did not generally impact the cognitive change measure which uses average decline across tasks in a cognitive index. However, for two patients, subsequent to the baseline session, motor dexterity declined secondary to osteoarthritis (n = 1) and SVD-related disability (n = 1) such that they were unable to use a pen/pencil for fine motor control and, as a result, it was not possible to assess performance for any of the tasks comprising the processing speed index. These patients were excluded from all analyses of processing speed.

## Results

### Demographics and patient flow

There were 98 participants in whom cognition was obtained on at least two time-points and data from these participants are used in this analysis. This excluded participants without any follow-up (n = 22), and those without neuropsychological follow-up (n = 1). Most participants (n = 65) had all four time-points of data, 17 had 3 time-points and 16 had 2 time-points. Fig 1 presents a flow diagram of patient status at each time-point. The median inter-test interval was 1 year, 5.25 days, and the median deviation from the 12 month target was +5.75 days, (25th percentile: −2.25 days, 75th percentile: +25.75 days).

Three patients suffered a new clinical stroke during the study. Two were subcortical lacunar strokes and one a small cortical hemorrhage. The protocol specified that patients with recurrent lacunar stroke remain within the study, but one of the two patients was unable to continue due to disability. The cortical hemorrhage was considered a study endpoint. Two further patients met study endpoints by converting to vascular dementia, defined by DSM-IV criteria. Neither case was associated with new clinical stroke. One further patient suffered brain ischemia associated with a persisting global cognitive deficit following a cardiac arrest and was excluded from later time-points.

Stated reasons for withdrawal (n = 15) included unwillingness due to poor health (n = 7), unwilling/study not worthwhile (n = 5), could not tolerate psychology (n = 1), could not tolerate MRI (n = 1) and no reason given (n = 1). Deaths (n = 12), where cause could be ascertained (n = 10), were not due to SVD-stroke (cancer–n = 2, cardiovascular–n = 2, respiratory–n = 1, other (health related)–n = 3, other (health unrelated)–n = 2). Sample sizes at each assessment were reduced by sporadic missed sessions (n = 12) due to ill health, scheduling issues or non-attendance. Furthermore, some patients attended MRI but not cognitive testing (n = 3) and others completed only the MMSE from the cognitive test battery (n = 2).

Baseline demographic and risk factor information is shown in Table 2 for both those included in the follow-up and those in whom follow-up cognition data was not available. Patients lacking any follow-up data tended to be older, with more severe disability and significantly poorer baseline cognitive function (Table 2).

**Table 2:** Demographic information for SVD cohort.

		Follow-up (n = 98)	Baseline only (n = 23)	Test Statistic
**Age (y)**		69.0 (9.93)	74.2 (7.76)	t_(40.8)_ = −2.7, p = 0.009
**Male Gender**		65 (66.3%)	13 (56.5%)	OR = 1.51, p = 0.5
**White Ethnicity**		69 (70.4%)	15 (65.2%)	OR = 0.79, p = 0.6
**Hypertension**		91 (92.9%)	21 (91.3%)	OR = 0.809, p = 0.7
**BP (Systolic) mmHg**		147.8 (21.61)	142.1 (20.71)	t_(28.2)_ = 1.1, p = 0.3
**BP (Diastolic) mmHg**		81.9 (10.7)	76.2 (9.93)	t_(28.9)_ = 2.3, p = 0.026
**Statin Therapy**		84 (85.7%)	19 (82.6%)	OR = 0.793, p = 0.7
**Rankin Score**	0	32 (32.7%)	6 (26.1%)	p = 0.037
	1	40 (40.8%)	8 (34.8%)	-
	2	14 (14.3%)	1 (4.3%)	-
	3	11 (11.2%)	5 (21.7%)	-
	4	1 (1%)	3 (13%)	-
**Diabetes**		19 (19.4%)	5 (21.7%)	OR = 1.15, p = 0.8
**Smoking**	Never	44 (44.9%)	11 (47.8%)	p = 0.8
	Ex	21 (21.4%)	3 (13%)	-
	Current	33 (33.7%)	9 (39.1%)	-
**Lacunar Strokes (prior to enrolment):**	One	75 (76.5%)	19 (82.6%)	p = 0.9
	Two	18 (18.4%)	3 (13%)	-
	>Two	5 (5.1%)	1 (4.3%)	-
Time to last stroke (weeks)*		26 (13, 156)	104 (16, 316)	Z = −1.82, p = 0.069
**Body Mass Index (kg/m2)**		27.0 (5.19)	27.1 (3.08)	t_(44.7)_ = −0.1, p = 0.9
**Baseline Cognition (Z score):**	WM	−0.13 (0.93)	−0.52 (0.72)	t_(41.1)_ = 2.2, p = 0.031
	LTM	0.059 (0.97)	−0.53 (0.90)	t_(35.2)_ = 2.8, p = 0.008
	PS	−0.74 (0.89)	−1.1 (1.00)	t_(28.5)_ = 1.6, p = 0.12
	EF	−0.77 (1.00)	−1.5 (0.91)	t_(34.6)_ = 3.4, p = 0.002
	Global	−0.49 (0.82)	−1.1 (0.8)	t_(33.8)_ = 3.0, p = 0.005

Values presented are Mean(SD) or N(%) unless indicated. Statistical tests presented are Welch’s t-tests for continuous variables and Fisher’s exact test for categorical data. Odds-ratios (OR) are displayed for 2x2 categorical data. BP—Blood pressure; WM—Working memory; LTM—Long-term (Episodic) memory; PS—Processing speed; EF—Executive function.

* Time to last stroke was non-normally distributed. For this measure Median (25^th^ percentile, 75^th^ percentile) values are presented and between-group differences are tested using an exact two sample Wilcoxon rank sum test[24].

### Baseline Cognition

At baseline SVD patients displayed impairments predominantly in the PS and EF indices consistent with previous reports[5,8]. The WM and LTM indices did not differ significantly from normal performance (Table 3). Male gender (p<0.02) and lower premorbid NART-IQ (p<0.0001) were associated with lower performance, but increased age was not (p = 0.69).

**Table 3:** Longitudinal analysis of cognition in SVD.

Measure		Baseline	Annualized Change
**Cognitive**	**Working Memory**	−0.13 (0.93), p = 0.177	−0.021 (0.37), p = 0.57
	**Episodic (Long Term) Memory**	0.059 (0.97), p = 0.55	0.062 (0.26), p = 0.02
	**Processing Speed**	−0.74 (0.89), p<2×10^−12^	−0.035 (0.23), p = 0.13
	**Executive Function**	−0.77 (1.00), p<8×10^−11^	−0.09 (0.32), p<0.007
	**Global**	−0.49 (0.82), p<5×10^−8^	−0.029 (0.20), p = 0.15
**Clinical**	**Rankin Scale Score**	1.39 (0.93)	0.43 (0.66), p<6×10^−9^

Table presents Mean (SD), and p-value from one-sample t-tests for cognitive indices and clinical measures.

#### Change in disability

There was a significant increase in disability, measured by the Rankin Scale[25], over the three year follow-up, with a mean (SD) annualised change of 0.43 (0.66; Table 3).

### Cognitive Change

As a preliminary step, task data were plotted and the model fits inspected to confirm the suitability of the annualized change measure. We further estimated an objective measure of model fit[26,27] (the Akaike information criterion with small sample correction) to compare linear measures of change with quadradic. These results are presented in S1 Table.

**Fig 2:**
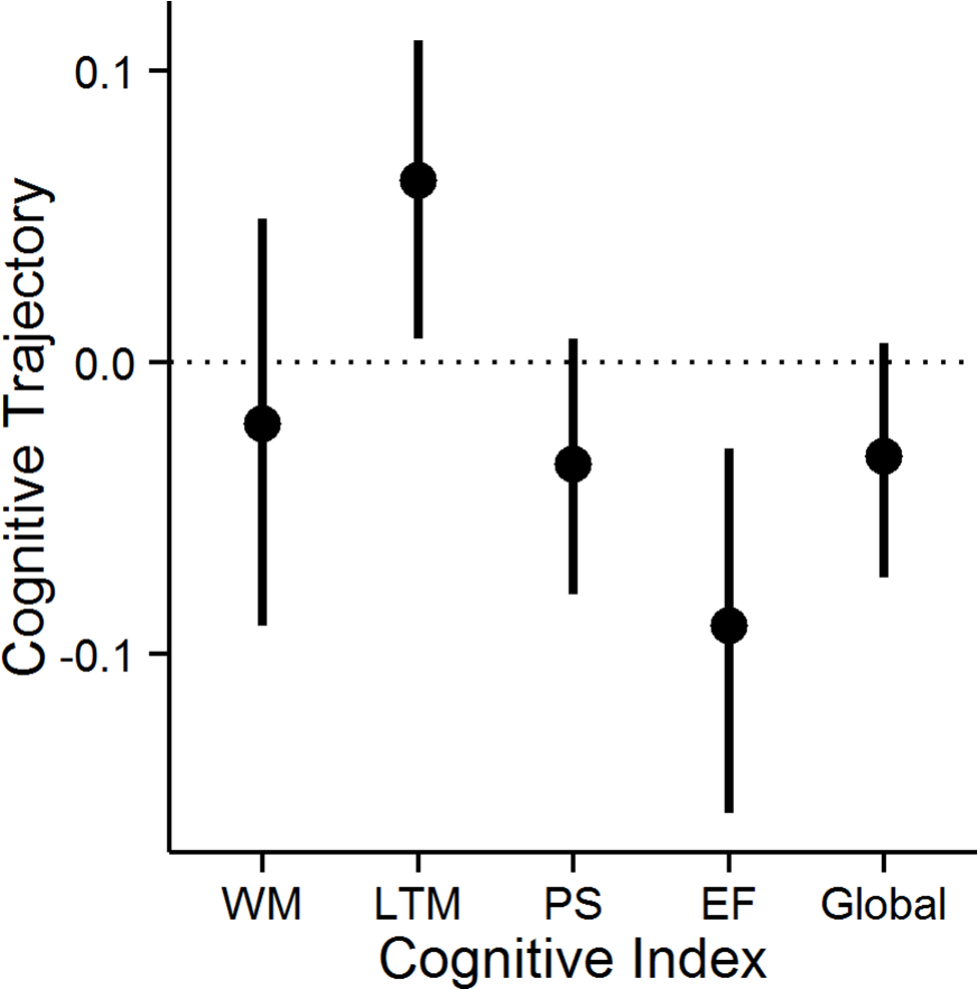
Estimates of annualized cognitive change cognitive indices. Mean annualized cognitive change (filled circles) are displayed for the four key cognitive indices: Working Memory (WM), Episodic (Long-term) Memory (LTM), Processing Speed (PS) and Executive Function (EF). The composite Global change is also displayed. Error bars show the 95% confidence interval of the mean obtained from non-parametric bootstrap estimation. The dotted line represents stable performance. Significant change occurs where error bars do not cross the dotted line.

**Fig 3:**
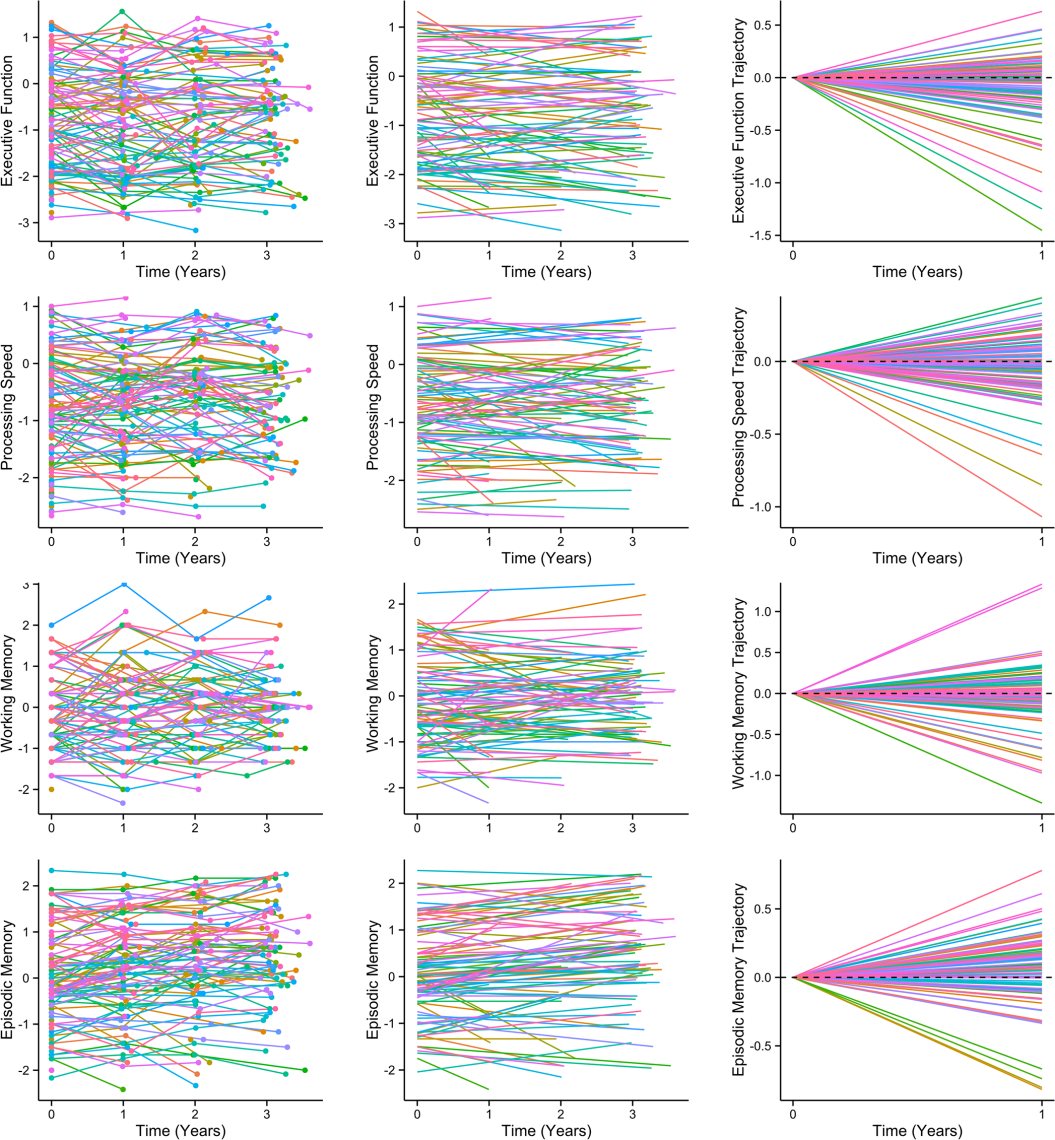
Cognitive change for the four cognitive indices. Rows present data for the four cognitive indices, from the top: 1^st^ row–Executive Function, 2^nd^ row–Processing Speed, 3^rd^ row–Working Memory, 4^th^ row–Episodic (long-term) memory. Left figures present ‘spaghetti-plots’ where the cognitive index scores at each assessment (circles) are presented with scores for each subject joined by lines. Middle column figures present the lines of best fit from the linear model. Right column figures show the annualized change measure: the trajectory of change over time from the linear model.

Analysis of the profile of cognitive change showed that the annualized rate of change was not equal across cognitive domains (F_(4,368)_ = 6.13, p<0.0001; Fig 2). Executive Function exhibited a significant decline (Table 3 & Fig 2). WM, PS and Global cognition showed a non-significant reduction over time. The LTM index improved over time, most likely reflecting a practice or learning effect. Age, gender and premorbid IQ did not explain variability in cognitive change: age (p = 0.99); gender (p = 0.66); NART-IQ (p = 0.83). There were also no significant interactions between these variables and the cognitive index factor: index×age (p = 0.82); index×gender (p = 0.41); index×NART-IQ (p = 0.81). All correlations between baseline cognitive performance on a measure and subsequent rate of cognitive change for that measure were non-significant: WM: r = −0.23, LTM: r = 0.098, PS: r = −0.081, EF: r = −0.105, Global: r = 0.11 (all p>0.1), as were inter-index correlations (absolute r ranged from 0.007 to 0.17; all p>0.1). Individual-level cognitive change data are plotted (Fig 3) for the four cognitive indices. Performance was heterogeneous and some subjects showed stable performance over the study.

### Sample size calculations

Table 4 compares the measures of cognition used in this study as potential endpoints in a future treatment study. Average 3-year difference scores are presented with the results of sample size calculations. The best performing individual tasks were single-letter verbal fluency (per arm n = 2,776, 30% effect) and the Wisconsin card sort task (n = 3,524, 30% effect). The multi-task composite EF index performed better than any single task (n = 2,192, 30% effect vs. n>2,776 for single tasks), an effect also seen in the PS composite (n = 7,475, 30% effect vs. n>9000 for single tasks). This may be attributed to some of the favorable properties of multi-task composite scores, which potentially allow performance to be assessed over a wider range of cognitive ability, and can diminish the impact of task-specific variability and ceiling/floor effects[28]. In this sample, floor level performances were commonly observed for several tasks, but especially the grooved pegboard (PS) and trail-making (EF) tests (GPT = 22.4%, TMT-B = 31.6%). In contrast, no subject performed at the minimum attainable level for any of the cognitive indices at baseline.

**Table 4:** Cognitive change and sample size estimates for tasks and composite indices.

		Mean (SD) Task Z-Scores			Sample-size Estimates			
Index	Task	Baseline	3-years	Difference	50%	40%	30%	20%
**Global**	(All)	−0.5 (0.83)	−0.6 (1.1)	−0.097 (0.61)	2,492	3,893	6,921	15,571
**WM**	DS	−0.13 (0.93)	−0.19 (1.3)	−0.064 (1.1)	19,027	29,730	52,858	>100k
**LTM**	Index	0.059 (0.97)	0.25 (1.3)	0.19 (0.78)	-	-	-	-
	LogMem-I	0.19 (1.1)	0.34 (1.5)	0.15 (1.1)	-	-	-	-
	LogMem-D	0.35 (1.1)	0.69 (1.5)	0.35 (1.1)	-	-	-	-
	VisRep-I	−0.48 (1.2)	−0.3 (1.5)	0.15 (1.2)	-	-	-	-
	VisRep-D	0.17 (1.1)	0.32 (1.5)	0.12 (1.2)	-	-	-	-
**PS**	Index	−0.74 (0.89)	−0.8 (1)	−0.1 (0.69)	2,692	4,205	7,475	16,818
	SOIP	−0.85 (0.93)	−0.87 (1.1)	−0.054 (0.71)	10,803	16,879	30,006	67,512
	GPT	−1.2 (1.4)	−1.3 (1.7)	−0.18 (1.3)	3,256	5,087	9,043	20,344
	DSST	−0.13 (0.87)	−0.15 (0.97)	−0.067 (0.62)	5,434	8,489	15,092	33,955
**EF**	EF-Index	−0.77 (1.0)	−1.0 (1.3)	−0.27 (0.96)	790	1,234	2,192	4,931
	SLVF	−0.2 (1.3)	−0.48 (1.6)	−0.28 (1.1)	1,000	1,562	2,776	6,245
	TMT-B	−1.2 (1.6)	−1.4 (2.0)	−0.15 (1.6)	6,465	10,101	17,957	40,402
	WCST	−0.9 (0.87)	−1.3 (1.6)	−0.38 (1.7)	1,269	1,983	3,524	7,928

Table presents mean (SD) Z-scores at baseline and 3-year follow-up and mean (SD) of their difference (follow-up − baseline) calculated per subject. Sample-size estimates show the number of patients per arm of a study required to detect a significant difference between groups equal to X% of the mean difference (alpha = 5%, power = 80%). Global—Global cognition index. WM—Working memory index. DS—Digit Span task. LogMem—Logical memory immediate (I) and delayed (D). VisRep—Visual reproduction task immediate (I) and delayed (D). LTM-Index—Long-term (Episodic) Memory Index. SOIP—BMIPB Speed of information processing task. GPT—Grooved pegboard task. DSST—Digit Symbol substitution task. PS-Index—Processing Speed index. SLVF—Single letter verbal fluency (FAS/BHR). TMT-B—Trail-making test-part B. WCST—Modified Wisconsin card sort test. EF-index—Executive function index.

## Discussion

During a three year follow-up study of cognition in patients with lacunar stroke and leukoaraiosis we report a pattern of change in cognition over time consistent with the baseline profile of impairments: average cognitive change declined most for the domains which were most impaired at baseline. However, cognitive change was variable and slow in most individuals such that measured declines were only statistically significant for executive function. The average rate of decline in executive function observed over 3-years in SVD is approximately a third of a standard deviation. This estimate in SVD is greater than comparable reports in healthy aging[29,30], but less than reported changes for general cognitive function, or memory scores, in both Alzheimer’s disease[31,32] and mild cognitive impairment[33].

Previous studies in SVD-related populations have investigated cognitive change in non-disabled individuals with radiological SVD (leukoaraiosis)[34], individuals with mixed vascular disease and vascular risk factors[35] and CADASIL[36,37]. In non-disabled individuals meeting radiological criteria for SVD[34] steeper 3-year cognitive decline than controls was reported for MMSE, verbal fluency (animal naming), Stroop color naming and trail making part A. No significant declines were found for immediate or delayed word recall, symbol digit modalities, digit span, trail making (B − A), or digit cancellation. Similarly, an older adult sample with vascular disease or risk factors[35] showed a 3-year decline on the symbol digit modalities test (comparable to the EF index in this sample), but no significant change in performance on a learning task. A large sample of CADASIL gene carriers followed up for an average of 24 months[36] showed significant decline in processing speed (trail making A) but not executive function (trail making B). In contrast, a small sample of CADASIL re-tested with a 7 year interval[37] showed significant decline on the CAMCOG battery (Global) and Stroop test (Executive), but not the trail-making task (Executive). In this group significant memory decline was also observed. Taken together with the results presented in our study from a clinically defined SVD population with radiological confirmation, there is significant task to task variability in decline over time in SVD, but more reliable decline in executive function tasks. It is an unanswered question whether the profile of decline is the same for CADASIL where samples span much larger age ranges.

Our results show that an executive function composite measure was the most sensitive to cognitive decline in this patient group, and that this was more sensitive than using individual cognitive tests. However, with any of the measures evaluated here, large sample sizes would be required to demonstrate the effectiveness of treatment aimed at halting cognitive decline. For example we estimate 2,192 patients in each arm would be required to detect a 30% reduction in the rate of EF cognitive decline during a three year follow-up. Even larger sample sizes would be required if other cognitive tests or domains were used. These numbers broadly agree with estimates from the LADIS study[38] where per-arm sample sizes required to detect a treatment effect over 3 years were estimated as 2,599 using the VADAS-cog battery and 1,809 using an EF composite measure. The large sample size estimates most likely reflect the slow and variable rate of decline in this patient group, although they may also be influenced by learning effects. Furthermore, looking at individual tasks, the better performing tasks were not those that SVD patients were most impaired on at baseline (trail-making, card sorting), but instead were tasks that showed smaller, but more reliable declines over the study period (single letter verbal fluency, digit symbol substitution). One explanation for this is the impact of performance floor effects for the former tests–individuals who perform at the lowest measurable level cannot subsequently decline regardless of their disease progress. These results suggest that future research into cognitive measures in SVD should focus on identifying executive function tasks which minimize practice effects and assess a wide range of deficit. However, the superior performance of composite measures coupled with the heterogeneity of change in SVD suggest that it may not be possible to have one single task to assess the condition. Finally, the large estimated sample sizes highlight the importance of alternative approaches to measuring cognitive function in treatment trials. One such approach is to use alternative markers to cognition, such as MRI measures[39] which have shown improved sensitivity to change in SVD[38]. A second approach is to improve sub-typing of SVD to identify those individuals likely to experience rapid cognitive decline.

This research should be interpreted with the following limitations in mind. First, no control group was included, so any cognitive decline could be caused by both SVD associated pathology and age related changes. However, previous case control studies have shown SVD is associated with impaired cognition compared with age matched normal control populations, and this has also been shown in the baseline data from the cohort we studied in this paper[5]. Second, sample size calculations were based on a hypothetical therapy which prevents cognitive decline subsequent to treatment. These calculations are not relevant where candidate therapies aim to treat existing cognitive impairments (i.e. to reverse cognitive decline), this is an aspect to be considered elsewhere.

In common with similar longitudinal studies[30,34,35,40], there was subject dropout, coupled with some systematic bias in the pattern of missing data. That is to say that despite considerable effort to assess all patients annually, a significant proportion did not complete the follow-up, and those who did not complete tended to be older, with greater disability and poorer cognition. Overall, the dropout rate was high (38%), and greater than similar studies of non-disabled elderly (e.g. LADIS[34]: 27%). As a result, the generalizability of our results may be reduced where substantially different patterns of withdrawal and/or loss to follow-up are obtained. This effect is likely to produce a modest underestimation of the average rate of cognitive change in SVD due to the effective censorship of data from those who decline the most, die, or withdraw from the research due to changed circumstances.

Furthermore, there are limitations related to the choice of cognitive measures. We selected measures that are widely used and that have previously been employed to investigate cognition in SVD[34,40,41], and we grouped task performance into cognitive domains. However neuropsychological tasks often lack complete specificity to any one cognitive domain and results should be consequently be interpreted with caution. Particularly, not all tasks controlled for motor impairment. As a result, some tasks contributing to the executive function, processing speed and episodic memory indices have significant motor performance elements rather than pure cognitive effects. Affected tasks include trail making, grooved pegboard, visual reproduction and digit symbol substitution. However, the decline seen in executive function factor is unlikely to be better explained by worsening motor speed deficits in SVD as the non-motor verbal fluency and card sorting tasks show larger declines. There were also practice effects present in the psychological data, most noticeably in the tasks assessing episodic memory where average performance increased over time. It is unclear the degree to which practice effects were present in other tasks and cognitive domains, however, where present, practice effects would also act to underestimate the degree of cognitive decline. The use of tasks with alternate forms, particularly for memory-based tasks would reduce this effect and so this is both a limitation of this research and a recommendation for future research. The learning effect for memory tasks highlights the preservation of memory in SVD and contrasts with the prominent decline reported in the AD/MCI phenotype[32,42]. Finally, cognitive trajectory is a simplistic model of change over time, but one that suits the characteristics of this data where task measures and inter-subject slopes are variable, and the participants who drop out tend to show the largest changes. With longer follow-up, more complex models of cognitive decline in SVD may be informative and particularly may be able to capture non-linear aspects of change.

In conclusion, in a group of patients with symptomatic SVD we found a significant decline in performance on executive function tasks over three years. In contrast, working memory and processing speed did not decline significantly, and practice effects were seen for episodic memory. An analysis of the sample size required for a treatment study established that large sample sizes would be needed to demonstrate the effectiveness of a treatment which acts to slow the rate of cognitive decline in SVD. These findings emphasise the importance of surrogate disease markers in SVD such as those provided by MRI.

## Supporting Information

S1 Table Descriptive statistics for Akaike information criterion. Descriptive statistics for the Akaike information criterion with small sample correction (AICc) are presented for linear and quadratic change in cognition for participants with complete data over all time points (n = 64). Smaller AICc values indicate superior fit. Mean average AICc values favor a linear fit over quadratic given the data.
(DOCX)

## References

[1] Pantoni L. Cerebral small vessel disease: from pathogenesis and clinical characteristics to therapeutic challenges. Lancet Neurol. 2010;9: 689–701. 10.1016/S1474-4422(10)70104-6 20610345

[2] Wardlaw JM, Smith C, Dichgans M. Mechanisms of sporadic cerebral small vessel disease: insights from neuroimaging. Lancet Neurol. 2013;12: 483–497. 10.1016/S1474-4422(13)70060-7 23602162 PMC3836247

[3] Charlton RA, Morris RG, Nitkunan A, Markus HS. The cognitive profiles of CADASIL and sporadic small vessel disease. Neurology. 2006;66: 1523–1526. 16717212 10.1212/01.wnl.0000216270.02610.7e

[4] Zhou A, Jia J. Different cognitive profiles between mild cognitive impairment due to cerebral small vessel disease and mild cognitive impairment of Alzheimer’s disease origin. J Int Neuropsychol Soc. 2009;15: 898–905. 10.1017/S1355617709990816 19891819

[5] Lawrence AJ, Patel B, Morris RG, MacKinnon AD, Rich PM, Barrick TR, et al Mechanisms of cognitive impairment in cerebral small vessel disease: multimodal MRI results from the St George’s cognition and neuroimaging in stroke (SCANS) study. PLoS One. 2013;8: e61014 10.1371/journal.pone.0061014 23613774 PMC3632543

[6] Mok VCT, Wong A, Lam WWM, Fan YH, Tang WK, Kwok T, et al Cognitive impairment and functional outcome after stroke associated with small vessel disease. J Neurol Neurosurg Psychiatry. 2004;75: 560–566. 15026497 10.1136/jnnp.2003.015107 PMC1739014

[7] Wardlaw JM, Smith EE, Biessels GJ, Cordonnier C, Fazekas F, Frayne R, et al Neuroimaging standards for research into small vessel disease and its contribution to ageing and neurodegeneration. Lancet Neurol. 2013;12: 822–838. 10.1016/S1474-4422(13)70124-8 23867200 PMC3714437

[8] Patel B, Lawrence AJ, Chung AW, Rich P, Mackinnon AD, Morris RG, et al Cerebral Microbleeds and Cognition in Patients With Symptomatic Small Vessel Disease. Stroke. 2013;44: 356–61. 10.1161/STROKEAHA.112.670216 23321452

[9] Lawrence AJ, Chung AW, Morris RG, Markus HS, Barrick TR. Structural network efficiency is associated with cognitive impairment in small-vessel disease. Neurology. 2014;83: 304–311. 10.1212/WNL.0000000000000612 24951477 PMC4115608

[10] Bamford J, Sandercock P, Dennis M, Burn J, Warlow C. Classification and natural history of clinically identifiable subtypes of cerebral infarction. Lancet. 1991;337: 1521–1526. 1675378 10.1016/0140-6736(91)93206-o

[11] Fazekas F, Chawluk JB, Alavi A, Hurtig HI, Zimmerman RA. MR signal abnormalities at 1.5 T in Alzheimer’s dementia and normal aging. Am J Roentgenol. 1987;149: 351–356. 3496763 10.2214/ajr.149.2.351

[12] Rankin J. Cerebral vascular accidents in patients over the age of 60. II. Prognosis. Scott Med J. 1957;2: 200–215. 13432835 10.1177/003693305700200504

[13] Coughlan AK, Oddy M, Crawford JR. The BIRT Memory and Information Processing Battery (B-MIPB). Wakefield, UK: The Brain Injury Rehabilitation Trust (BIRT); 2007.

[14] Nelson H, Willison JR. National Adult Reading Test (NART): Test Manual. 2nd ed. 1991.

[15] Wechsler D. Wechsler Adult Intelligence Scale-Third edition (WAIS-III). San Antonio, TX: The Psychological Corporation; 1997.

[16] Wechsler D. Wechsler Memory Scale—Third Edition (WMS-III UK) Administration and Scoring Manual. San Antonio, TX: The Psychological Corporation; 1997.

[17] Matthews CG, Klove H. Instruction manual for the Adult Neuropsychology Test battery. University of Wisconsin Medical School; 1964.

[18] Dawson JD, Uc EY, Anderson SW, Johnson AM, Rizzo M. Neuropsychological predictors of driving errors in older adults. J Am Geriatr Soc. 2010;58: 1090–1096. 10.1111/j.1532-5415.2010.02872.x 20487082 PMC3204878

[19] Reitan RM. The relation of the trail making test to organic brain damage. J Consult Psychol. 1955;19: 393–394. 13263471 10.1037/h0044509

[20] Mitrushina M, Boone KB, Razani J, D’Elia LF. Handbook of Normative Data for Neuropsychological Assessment. 2nd ed. USA: Oxford University Press; 2005.

[21] Delis DC, Kaplan E, Kramer JH. Delis-Kaplan Executive Function Scale (D-KEFS). San Antonio, TX: The Psychological Corporation; 2001.

[22] Nagahama Y, Okina T, Suzuki N, Matsuzaki S, Yamauchi H, Nabatame H, et al Factor structure of a modified version of the wisconsin card sorting test: an analysis of executive deficit in Alzheimer’s disease and mild cognitive impairment. Dement Geriatr Cogn Disord. 2003;16: 103–112. 10.1159/000070683 12784035

[23] R Development Core Team. R: A Language and Environment for Statistical Computing [Internet]. Vienna, Austria; 2011. Available: http://www.R-project.org/

[24] Hothorn T, Hornik K, Wiel MA van de, Zeileis A. Implementing a Class of Permutation Tests: The coin Package. J Stat Softw. 2008;28: 1–23.27774042

[25] Van Swieten JC, Koudstaal PJ, Visser MC, Schouten HJ, van Gijn J. Interobserver agreement for the assessment of handicap in stroke patients. Stroke. 1988;19: 604–607. 3363593 10.1161/01.str.19.5.604

[26] Akaike H. A new look at the statistical model identification. IEEE Trans Autom Control. 1974;19: 716–723. 10.1109/TAC.1974.1100705

[27] Burnham KP, Anderson DR. Multimodel Inference Understanding AIC and BIC in Model Selection. Sociol Methods Res. 2004;33: 261–304. 10.1177/0049124104268644

[28] Crawford JR. Psychometric Foundations of Neuropsychological Assessment In: Goldstein LH, McNeil JE, editors. Clinical Neuropsychology: A Practical Guide to Assessment and Management for Clinicians. John Wiley & Sons, Ltd; 2003 pp. 121–140.

[29] Garde E, Lykke Mortensen E, Rostrup E, Paulson OB. Decline in intelligence is associated with progression in white matter hyperintensity volume. J Neurol Neurosurg Psychiatry. 2005;76: 1289–1291. 10.1136/jnnp.2004.055905 16107370 PMC1739790

[30] Charlton RA, Barrick TR, McIntyre DJ, Shen Y, O’Sullivan M, Howe FA, et al White matter damage on diffusion tensor imaging correlates with age-related cognitive decline. Neurology. 2006;66: 217–222. 16434657 10.1212/01.wnl.0000194256.15247.83

[31] Haxby JV, Raffaele K, Gillette J, Schapiro MB, Rapoport SI. Individual trajectories of cognitive decline in patients with dementia of the Alzheimer type. J Clin Exp Neuropsychol. 1992;14: 575–592. 10.1080/01688639208402846 1400920

[32] Wilson RS, Segawa E, Boyle PA, Anagnos SE, Hizel LP, Bennett DA. The Natural History of Cognitive Decline in Alzheimer’s Disease. Psychol Aging. 2012;27: 1008–1017. 10.1037/a0029857 22946521 PMC3534850

[33] Lim YY, Maruff P, Pietrzak RH, Ellis KA, Darby D, Ames D, et al Aβ and cognitive change: Examining the preclinical and prodromal stages of Alzheimer’s disease. Alzheimers Dement J Alzheimers Assoc. 2014;Epub. 10.1016/j.jalz.2013.11.005 24589436

[34] Jokinen H, Kalska H, Ylikoski R, Madureira S, Verdelho A, van der Flier WM, et al Longitudinal cognitive decline in subcortical ischemic vascular disease–the LADIS Study. Cerebrovasc Dis. 2009;27: 384–391. 10.1159/000207442 19276621

[35] Van den Heuvel DMJ, ten Dam VH, de Craen AJM, Admiraal-Behloul F, Olofsen H, Bollen ELEM, et al Increase in periventricular white matter hyperintensities parallels decline in mental processing speed in a non-demented elderly population. J Neurol Neurosurg Psychiatry. 2006;77: 149–153. 10.1136/jnnp.2005.070193 16421114 PMC2077562

[36] Jouvent E, Mangin J-F, Duchesnay E, Porcher R, Düring M, Mewald Y, et al Longitudinal changes of cortical morphology in CADASIL. Neurobiol Aging. 2012;33: 1002.e29–1002.e36. 10.1016/j.neurobiolaging.2011.09.013 22000857

[37] Liem MK, Lesnik Oberstein SAJ, Haan J, van der Neut IL, Ferrari MD, van Buchem MA, et al MRI correlates of cognitive decline in CADASIL: a 7-year follow-up study. Neurology. 2009;72: 143–148. 10.1212/01.wnl.0000339038.65508.96 19139365

[38] Schmidt R, Berghold A, Jokinen H, Gouw AA, van der Flier WM, Barkhof F, et al White matter lesion progression in LADIS: frequency, clinical effects, and sample size calculations. Stroke J Cereb Circ. 2012;43: 2643–2647. 10.1161/STROKEAHA.112.662593 22879094

[39] Patel B, Markus HS. Magnetic resonance imaging in cerebral small vessel disease and its use as a surrogate disease marker. Int J Stroke. 2011;6: 47–59. 10.1111/j.1747-4949.2010.00552.x 21205241

[40] Sachdev PS, Brodaty H, Valenzuela MJ, Lorentz LM, Koschera A. Progression of cognitive impairment in stroke patients. Neurology. 2004;63: 1618–1623. 15534245 10.1212/01.wnl.0000142964.83484.de

[41] Nitkunan A, Barrick TR, Charlton RA, Clark CA, Markus HS. Multimodal MRI in cerebral small vessel disease: its relationship with cognition and sensitivity to change over time. Stroke. 2008;39: 1999–2005. 10.1161/STROKEAHA.107.507475 18436880

[42] Mura T, Proust-Lima C, Jacqmin-Gadda H, Akbaraly TN, Touchon J, Dubois B, et al Measuring cognitive change in subjects with prodromal Alzheimer’s disease. J Neurol Neurosurg Psychiatry. 2014;85: 363–370. 10.1136/jnnp-2013-305078 23840054 PMC5225268

