# Supplementary material for: Pattern and Rate of Cognitive Decline in Cerebral Small Vessel Disease: A Prospective Study
Source: PLoS One. 2015 Aug 14;10(8):e0135523. doi: 10.1371/journal.pone.0135523 (PMC4537104; doi:10.1371/journal.pone.0135523)
Supplement: S1 Table — Descriptive statistics for the Akaike information criterion with small sample correction (AICc) are presented for linear and quadratic change in cognition for participants with complete data over all time points (n = 64). Smaller AICc values indicate superior fit. Mean average AICc values favor a linear fit over quadratic given the data. (DOCX) [file pone.0135523.s001.docx]

**S1 Table. Descriptive statistics for Akaike information criterion.**

|  | AICc Linear | AICc Quadratic |
| --- | --- | --- |
| Mean | 7.95 | 14.53 |
| SD | 5.71 | 9.08 |
| Min | -24.29 | -28.30 |
| Max | 21.71 | 33.44 |

Descriptive statistics for the Akaike information criterion with small sample correction (AICc) are presented for linear and quadratic change in cognition for participants with complete data over all time points (n=64). Smaller AICc values indicate superior fit. Mean AICc values favor a linear fit over quadratic given the data.
